# Development and validation of a survival model for esophageal adenocarcinoma based on autophagy-associated genes

**DOI:** 10.1080/21655979.2021.1946235

**Published:** 2021-07-12

**Authors:** Lili Duan, Lu Cao, Rui Zhang, Liaoran Niu, Wanli Yang, Weibo Feng, Wei Zhou, Junfeng Chen, Xiaoqian Wang, Yiding Li, Yujie Zhang, Jinqiang Liu, Qingchuan Zhao, Daiming Fan, Liu Hong

**Affiliations:** a Division of Digestive Surgery, State Key Laboratory of Cancer Biology and National Clinical Research Center for Digestive Diseases, Xijing Hospital of Digestive Diseases, Fourth Military Medical University, Xi’an, Shaanxi Province, China; b Department of Biomedical Engineering, Fourth Military Medical University, Xi’an, Shaanxi Province, China

**Keywords:** Esophageal adenocarcinoma, autophagy, prognosis, nomogram, bioinformatics

## Abstract

Autophagy is a highly conserved catabolic process which has been implicated in esophageal adenocarcinoma (EAC). We sought to investigate the biological functions and prognostic value of autophagy-related genes (ARGs) in EAC. A total of 21 differentially expressed ARGs were identified between EAC and normal samples. Gene Ontology (GO) and Kyoto Encyclopedia of Genes and Genomes (KEGG) analysis were then applied for the differentially expressed ARGs in EAC, and the protein–protein interaction (PPI) network was established. Cox survival analysis and Lasso regression analysis were performed to establish a prognostic prediction model based on nine overall survival (OS)-related ARGs (CAPN1, GOPC, TBK1, SIRT1, ARSA, BNIP1, ERBB2, NRG2, PINK1). The 9-gene prognostic signature significantly stratified patient outcomes in The Cancer Genome of Atlas (TCGA)-EAC cohort and was considered as an independently prognostic predictor for EAC patients. Moreover, Gene set enrichment analysis (GSEA) analyses revealed several important cellular processes and signaling pathways correlated with the high-risk group in EAC. This prognostic prediction model was confirmed in an independent validation cohort (GSE13898) from The Gene Expression Omnibus (GEO) database. We also developed a nomogram with a concordance index of 0.78 to predict the survival possibility of EAC patients by integrating the risk signature and clinicopathological features. The calibration curves substantiated favorable concordance between actual observation and nomogram prediction. Last but not least, Erb-B2 Receptor Tyrosine Kinase 2 (ERBB2), a member of the prognostic gene signature, was identified as a potential therapeutic target for EAC patients. To sum up, we established and verified a novel prognostic prediction model based on ARGs which could optimize the individualized survival prediction in EAC.

## graphical

**Figure:**
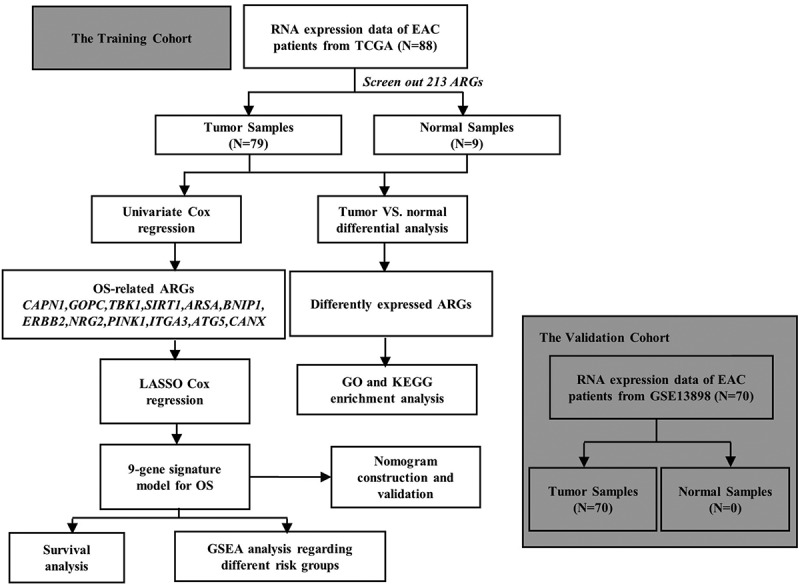

## Highlights

1. A 9-autophagy-associated gene signature was developed to predict the EAC patient prognosis.

2. GSEA analysis showed potential molecular functions and mechanisms of 9-ARGs signature in EAC.

3. A nomogram was established to predict the survival possibility of EAC patients by integrating the risk signature and clinicopathological features.

## Introduction

Esophageal cancer (EC) is a common malignant cancer worldwide, ranking the seventh in terms of incidence and sixth in terms of causes of cancer-related death [1]. Histologically, EC is classified into two main types: esophageal squamous cell carcinoma (ESCC) and esophageal adenocarcinoma (EAC), which have quite different etiologies. Interestingly, EAC represents the majority of EC cases in developed countries and its incidence rate rises dramatically on account of excess body weight, increasing gastroesophageal reflux disease (GERD), and tobacco smoking [2]. The development of EAC was characterized by a gradual transformation process from high-grade dysplasia to metaplastic Barrett’s esophagus (BE), and ultimately to invasive carcinoma [3]. Despite great progress has been made in therapeutic methods and medical management, many EAC patients suffered from diagnosed at advanced stage and poor prognosis [4]. Therefore, it is imperative to develop effective prognostic biomarkers to optimize the treatment of EAC patients.

Autophagy refers to a highly conserved and tightly regulated cellular catabolic event for degrading and recycling cellular components, such as aggregated proteins and damaged organelles [5]. The dysregulation of autophagy is closely associated with multiple diseases, such as neurodegenerative diseases, immune disorders, and cancers [6]. Multiple signaling transduction pathways known to regulate critical cellular processes are also implicated in autophagy regulation, including PI3K/AKT/mTOR, RAS, JAK-STAT, AMPK/CaMKK, and p53/DRAM signaling pathways [7]. Mounting evidences have revealed the implication of autophagy in EAC [8]. Autophagy plays an intricate role in esophageal carcinogenesis. Previous study reported that autophagy functioned to decrease reactive oxygen species (ROS) levels and dispose the ROS-mediated organelle damage following acid exposure in BE, while excessive ROS contributed to foster neoplastic transformation [9]. Notably, the decreased autophagy following chronic exposure to bile acids was closely associated with increased genomic instability and EAC progression [10]. Recent studies have shown that autophagy was also involved in 5-fluorouracil (5-FU) and cisplatin acquired resistance in EAC [11]. Moreover, tumors with low expression of autophagy markers p62 and LC3B exhibited more aggressive behaviors and correlated with worse prognosis in EAC [12]. These findings confirm the involvement of autophagy in EAC and indicate that ARGs may have great potential as prognostic biomarkers in EAC.

Nevertheless, autophagy is an intricate process involving a number of molecules. Therefore, a prognostic gene signature integrating multiple ARGs may improve the accuracy of prognosis prediction in contrast with the single gene. We have thoroughly reviewed several other papers which have examined the prognostic significance of the ARGs in esophageal cancer. Firstly, a 7-gene model (TBK1, ATG5, HSP90AB1, VAMP7, DNAJB1, GABARAPL2, and MAP2K7) was constructed by Chen C et.al. with the area under curve (AUC) value of >0.6 [13]. And Hailei Du et al. established a 4-gene model (DNAJB1, BNIP1, VAMP7, and TBK1), which significantly divided ESCA patients into high- and low-risk groups in terms of OS [hazard ratio (HR) = 1.508, 95% confidence interval (CI): 1.201–1.894, P < 0.001] [14]. However, these two studies failed to separate out adenocarcinoma and squamous carcinoma. Given the different etiologies of adenocarcinoma and squamous carcinoma, it is essential to develop a prediction model in the specific type of esophageal cancer. Another two studies which were conducted by Zhu L et.al [15] and Xu T et al [16] focused only on adenocarcinoma. Although different analysis methods were adopted in these two studies, the final prognostic models were identical. Unfortunately, the above four papers all failed to validated their final model in the independent dataset. In addition, despite the gene of interest were screened using different methods, the final models were all established using MultiCox regression in the above four studies. However, according to the issue proposed by Huang Y, et al. [17]. The number of events should exceed the number of included covariates by at least 10 times in a multivariate analysis. Therefore, the least absolute shrinkage and selection operator (LASSO) Cox regression model, which is suitable for the regression of high-dimensional data, was used to select the most useful prognostic features in the training data set in our study. The detailed comparisons among these four studies and our study were shown in Table S1.

In this study, we focused on investigating the associations between ARGs profiles and prognosis of EAC patients. We developed a novel scoring system based on 9-ARGs signature that was identified as an independently prognosis predictor for EAC patients. Our findings might contribute to further understand the functional role of ARGs in EAC pathogenesis and to improve clinical practice.

## Materials and methods

### Data acquisition and pre‑processing

The entire set of 232 ARGs was obtained from the Human Autophagy-dedicated Database (HADb) (http://www.autophagy.lu/index.html). RNA sequencing data and clinical features consisting of 79 EAC and 9 non-tumor tissues was downloaded from the TCGA data portal (https://portal.gdc.cancer.gov/). R software (version 3.6.3) was utilized to normalize and process data.

The Gene Expression Omnibus (GEO) database was applied to screen independent cohorts for the validation of the prognostic prediction model (https://www.ncbi.nlm.nih.gov/geo/). Combined the search term ‘esophageal adenocarcinoma’ and study type ‘Expression profiling by array’, the GSE13898 dataset (https://www.ncbi.nlm.nih.gov/geo/query/acc.cgi?acc=GSE13898) with complete clinicopathological data was identified as the uniquely applicable candidate cohort for the validation study.

### Identification of the differentially expressed ARGs

The package ‘limma’ in R software was used to identify the differentially expressed ARGs between EAC and their non-tumor counterparts, with thresholds of |log2 fold change (FC)|> 1.5 and FDR < 0.05. Then, we use online tool STING (https://string-db.org/cgi/input.pl) to establish the protein–protein interaction (PPI) network of the differentially expressed ARGs.

### Identification and validation of the prognostic prediction model based on ARGs

Univariate Cox and multivariate Cox regression analyses were carried out to screen the overall survival (OS)-related ARGs in the TCGA EAC dataset. Then, the OS-related ARGs were subjected to LASSO COX regression analysis to establish the prognostic prediction model using the R package ‘glmnet’. The following formula was applied to calculate the risk score for each patient:$riskscore=\overset{n}{\underset{j=1}{\sum }}Coefj*Xj$, with Coef_j_ representing the coefficient and X_j_ indicating the relative expression level of each ARG standardized by z-score. The median risk score was set as a cutoff value to divide the TCGA-EAC cohort into high and low-risk group. Kaplan–Meier method was conducted to evaluate differences between high and low-risk group in terms of survival. In addition, the receiver-operator characteristic (ROC) curve was drawn to assess the accuracy of prognostic prediction model using R package ‘survivalROC’. The impact of each gene in the prognostic prediction model on EAC survival was evaluated via the online Kaplan–Meier Plotter tool (http://kmplot.com/analysis/). Furthermore, the prognostic prediction model was verified in an independent EAC cohort (GSE13898).

### Development of nomogram

Risk score and clinicopathological factors (age, gender, T, N, M, and stage) were integrated to construct a nomogram for predicting 1- and 3-year survival possibility of EAC patients, using the R ‘survival’ and ‘rms’ packages. Moreover, calibration curves were plotted to evaluate the consistency between predicted and actual survival.

### Functional enrichment analysis

Gene Ontology (GO) and Kyoto Encyclopedia of Genes and Genomes (KEGG) enrichment analysis were then performed to explore the potential function and signaling pathways related to the differentially expressed ARGs in EAC using the R package ‘clusterprofiler’.

Gene Set Enrichment Analysis (GSEA) (http://software.broadinstitute.org/gsea/) was conducted to assess biological processes (BP), cellular components (CC), molecular functions (MF), and the signaling pathways enrichment between high-risk and low-risk group. p < 0.05 and false discovery rate (FDR) FDR < 0.25 were defined as the cutoff values.

### Mutation, CNV, Methylation, and Regulatory Networks Analysis of ERBB2

The UCSC Xena browser (https://xenabrowser.net/heatmap/), the cBio Cancer Genomics Portal (http://www.cbioportal.org), cancer cell line encyclopedia (CCLE) database (https://portals.broadinstitute.org/ccle) were applied to explore the relationship between ERBB2 mRNA expression, copy number variation (CNV), somatic mutation, and DNA methylation.

Transcription factors (TFs) and gene target data derived from the ENCODE ChIP-seq data were utilized to predict translational factors for ERBB2 (https://www.enco-deproject.org/). The interactions between microRNAs (miRNAs) and ERBB2 were obtained based on the data provided by Tarbase (http://diana.cslab.ece.ntua.gr/tarbase/) and miRTarBase (http://mirtarbase.mbc.nctu.edu.tw/php/index.php).

### Statistical analysis

All statistical analyses including univariate and multivariate Cox regression analyses, LASSO regression analysis, Kaplan–Meier survival analyses and ROC curve analysis were executed using the R software 3.6.3 and GraphPad Prism 7 (San Diego, CA, USA). P < 0.05 was set as statistical significance for above analysis.

## Results

In the present study, we investigated the correlation between expression profiles of ARGs and prognosis of EAC patients. Totally 21 differentially expressed ARGs were screened between EAC and adjacent non-tumor samples in TCGA EAC cohort. Gene Ontology (GO) and Kyoto Encyclopedia of Genes and Genomes (KEGG) analysis were conducted to explore the potential roles of the differentially expressed ARGs in EAC. Cox proportional hazard regression analysis was performed to screen prognosis-associated genes out of 213 ARGs in The Cancer Genome of Atlas (TCGA) EAC cohort. And then, the generated genes were subjected to LASSO to construct an optimal model of prognostic prediction, followed by validation in an independent GEO EAC cohort. Patients were separated into high-risk and low-risk groups based on the median risk score. The Kaplan–Meier survival curves and the receiver operating characteristic (ROC) curves were plotted to evaluate the performance of the model. Gene Set Enrichment Analysis (GSEA) was applied to explore the differences between high and low-risk groups. Furthermore, we established a nomogram to predict the EAC patients’ survival probability by integrating the risk signature and clinicopathological factors. Finally, among the prognostic gene signature, Erb-B2 Receptor Tyrosine Kinase 2 (ERBB2) was identified as a potential therapeutic target for EAC patients.

### Characteristics of patients

EAC cohorts in TCGA database consisted of a total of 79 esophageal adenocarcinoma patients. The clinical features of EAC patients were listed in Table S2. Kaplan–Meier survival curves of tumor (T), lymph (N), metastasis (M), and stage were plotted for EAC cohorts (Figure S1).

### Differentially expressed autophagy-associated genes between EAC and adjacent non-tumor tissues

**Figure 1.**
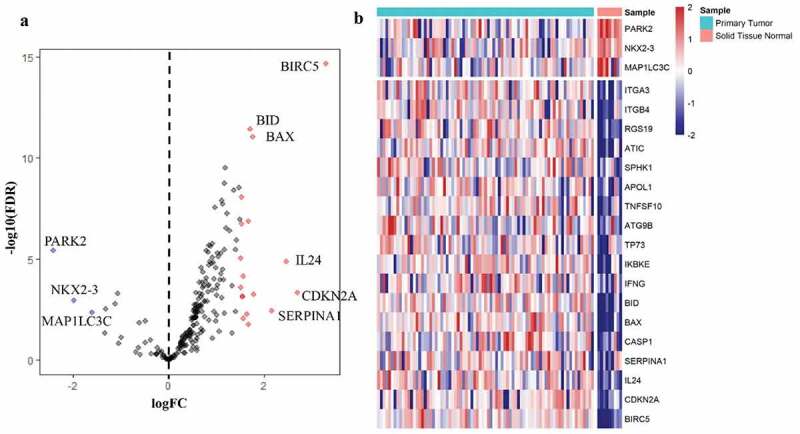
**Differentially expressed autophagy-associated genes in esophageal adenocarcinoma (EAC) and non-tumor samples**. (a) The volcano map of 213 autophagy-associated genes. The red dots indicate upregulated genes and the blue dots represent downregulated genes. (b) Hierarchical clustering distribution of differentially expressed autophagy-associated genes in EAC tissues and normal tissues

A total of 232 ARGs were obtained from the HADB database, 213 of which were expressed in TCGA EAC dataset. The expression of 213 ARGs was analyzed in 79 EAC and 9 non-tumor tissues using ‘limma’ package in R, and 21 differentially expressed ARGs were eventually screened with the criteria of |log2FC| > 1.5 and FDR < 0.05, including 18 upregulated genes (BIRC5, CDKN2A, IL24, SERPINA1, CASP1, BAX, BID, IFNG, IKBKE, TP73, ATG9B, TNFSF10, APOL1, SPHK1, ATIC, RGS19, ITGB4, ITGA3) and 3 downregulated genes (MAP1LC3C, NKX2-3, PARK2) (Figure 1a-b). Then, these differentially expressed ARGs were subjected to STRING to construct a protein–protein interaction (PPI) network to understand the interaction among the differentially expressed ARGs, as shown in Figure S2.

### GO and KEGG analyses of autophagy-associated genes

**Figure 2.**
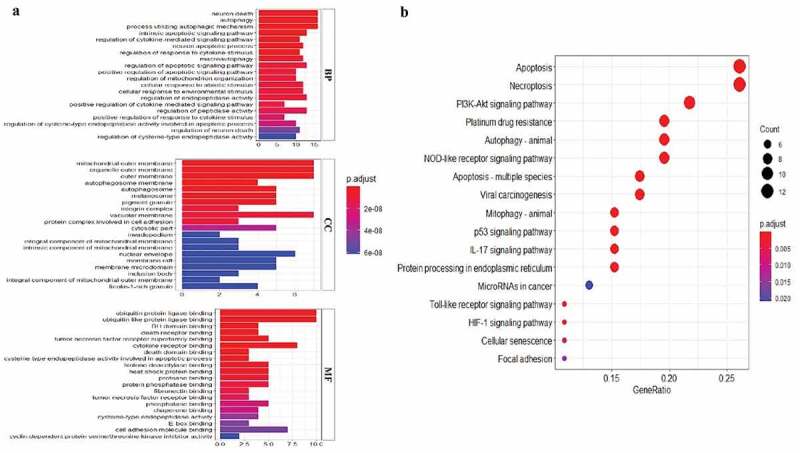
**GO and KEGG enrichment analysis**. (a) GO analysis of 21 differentially expressed autophagy-related genes. ‘BP’ represents ‘biological process’, ‘CC’ represents ‘cellular component’ and ‘MF’ represents ‘molecular function’. (b) KEGG analysis of 21 differentially expressed autophagy-related genes

To explore the potential function and signaling pathways related to the differentially expressed ARGs in esophageal adenocarcinoma, GO functional annotation and KEGG pathway enrichment analyses were applied (Figure 2a, b).

GO analysis indicated that these differentially expressed ARGs could be categorized into several BP, including autophagy, intrinsic apoptotic signaling pathway, and neuron death (Figure 2a). The top enriched CC terms related to differentially expressed ARGs were mitochondrial outer membrane, autophagosome, and nuclear envelope (Figure 2a). The most enriched MF terms of differentially expressed ARGs were ubiquitin protein ligase binding, cytokine receptor binding and cell adhesion molecule binding (Figure 2a). KEGG analysis showed that differentially expressed ARGs were mainly correlated with autophagy, apoptosis, PI3K-AKT signaling pathway, platinum drug resistance, and p53 signaling pathway (Figure 2b).

### Identification of prognosis‑related ARGs and construction of prognostic prediction model

**Figure 3.**
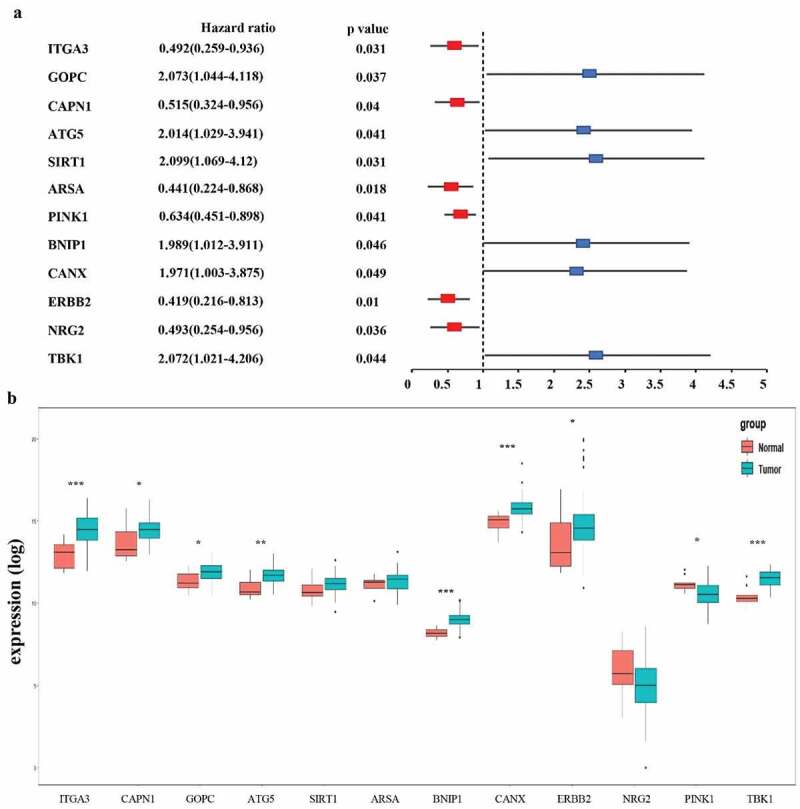
**Selection of autophagy genes associated with the survival of esophageal adenocarcinoma by univariate Cox regression analysis**. (a) Forest plot of autophagy genes associated with TCGA-EAC survival. (b) Differential expression of the 12 selected genes between normal and EAC tissues

All 213 ARGs were subjected to univariate Cox regression analysis. A total of 12 ARGs were significantly correlated with the OS of TCGA-EAC (Figure 3a, b). Among significant genes, eight genes (SIRT1, GOPC, TBK1, MAPK9, ATG5, BNIP1, CANX) were identified as risk factors and their overexpression were associated with worse outcome, whereas overexpression of the NRG2, ITGA3, ARSA, and ERBB2 may predict favorable outcome in EAC patients.

**Figure 4.**
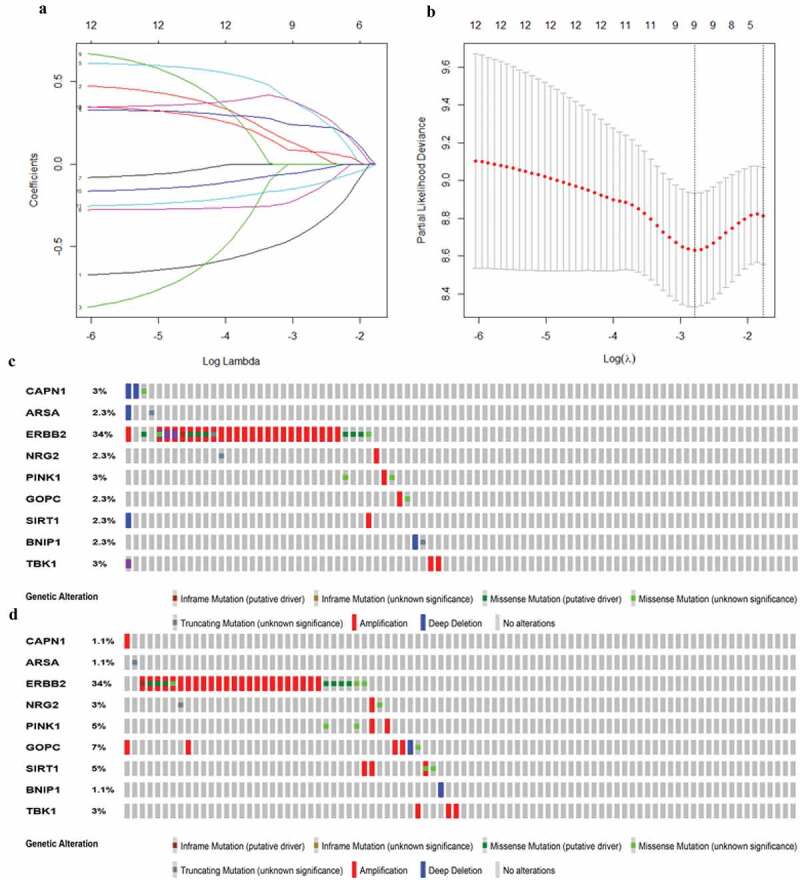
**Establishment of prognostic gene signature by LASSO regression analysis**. (a) Lasso coefficient profiles of the 12 autophagy-associated genes in EAC. (b) The optimal lambda value in Lasso model. (c) Genetic alteration of 9 autophagy-associated genes in the ESCA cohort (TCGA, PanCancer Atlas). (d) Genetic alteration of 9 autophagy-associated genes in the ESCA cohort (TCGA, Firehose Legacy)

Then, all these survival-related ARGs were subjected to LASSO COX regression analysis. The regression coefficient of each gene in EAC was illustrated in Figure 4a. As shown in Figure 4b, the model achieved the best performance when nine genes (CAPN1, GOPC, TBK1, SIRT1, ARSA, BNIP1, ERBB2, NRG2, PINK1) were included. Table 1 was drawn to show the coefficients and functions of these genes, which mainly correlated with facilitating the formation of autophagosomes, autophagosome maturation, as well as apoptosis regulation.

**Table 1.** Functions of genes in the prognostic gene signatures

No	Gene symbol	Full name	Function	Risk coefficient
1	CAPN1	Calpain 1	Play a role in the initiation of autophagy	−0.3070954
2	ARSA	Arylsulfatase A	Involved in regulation of autophagy	−0.2100786
3	ERBB2	Erb-B2 Receptor Tyrosine Kinase 2	Regulate autophagy and apoptosis	−0.1178015
4	NRG2	Neuregulin 2	Play a role in autophagy	−0.1494456
5	PINK1	PTEN Induced Kinase 1	Involved in the clearance of damaged mitochondria via selective autophagy (mitophagy)	−0.1800211
6	GOPC	Golgi Associated PDZ And Coiled-Coil Motif Containing	play a role in autophagy	0.2283383
7	SIRT1	Sirtuin 1	Participate in the coordination of apoptosis and autophagy	0.3195141
8	BNIP1	BCL2 Interacting Protein 1	Involved in mitochondrial autophagy and apoptosis	0.1733898
9	TBK1	TANK Binding Kinase 1	Promoting autophagosome maturation	0.3517779

For an in‐depth knowledge of the contributions of the above 9 survival-related ARGs to esophageal carcinogenesis, the cBio Cancer Genomics Portal was performed to investigate the genetic alteration of these genes (http://www.cbioportal.org). Datasets of Firehose Legacy and PanCancer Atlas for EAC were applied (Esophageal Adenocarcinoma: 88 samples in Firehose Legacy vs. 87 samples in PanCancer Atlas). Genes of interest were altered in 41 (47%) of 87 queried patients (PanCancer Atlas) (Figure 4c), compared with that altered queried genes were detected in 44 (50%) of 88 sequenced patients (Firehose Legacy) (Figure 4d). The frequent genetic alterations indicated the pivotal roles of these genes in esophageal carcinogenesis.

**Figure 5.**
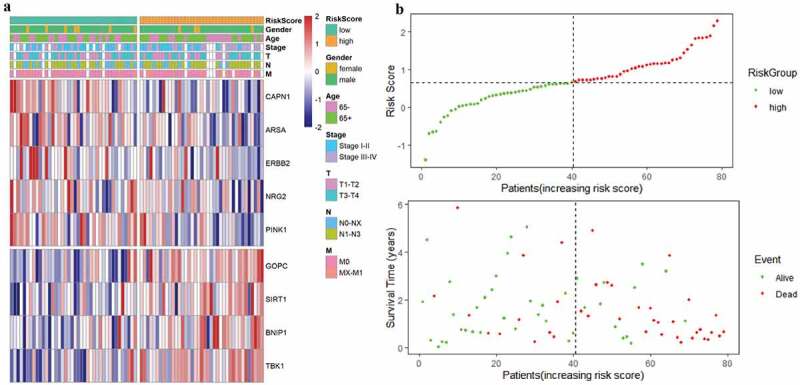
**Characteristics of the prognostic gene signature**. (a) Heatmap of the autophagy-associated gene expression profiles in prognostic signature for TCGA-EAC. (b) The distribution of risk score and patient’s survival time, as well as status for TCGA-EAC

The risk score of each EAC patient was calculated formulated on the risk coefficients and mRNA expression levels of selected genes. The risk score was used to predict the prognosis of EAC patients, and the median risk score was applied as a cutoff value to divide patients into low-risk and high-risk groups. A heatmap was plotted to exhibit the gene expression profiles in low-risk and high-risk EAC groups (Figure 5a). Genes (CAPN1, ARSA, ERBB2, NRG2, and PINK1) with HR<1 were identified as protective genes, while those (GOPC, SIRT1, BNIP1, and TBK1) with HR>1 as risk genes (Figure 5a).

**Figure 6.**
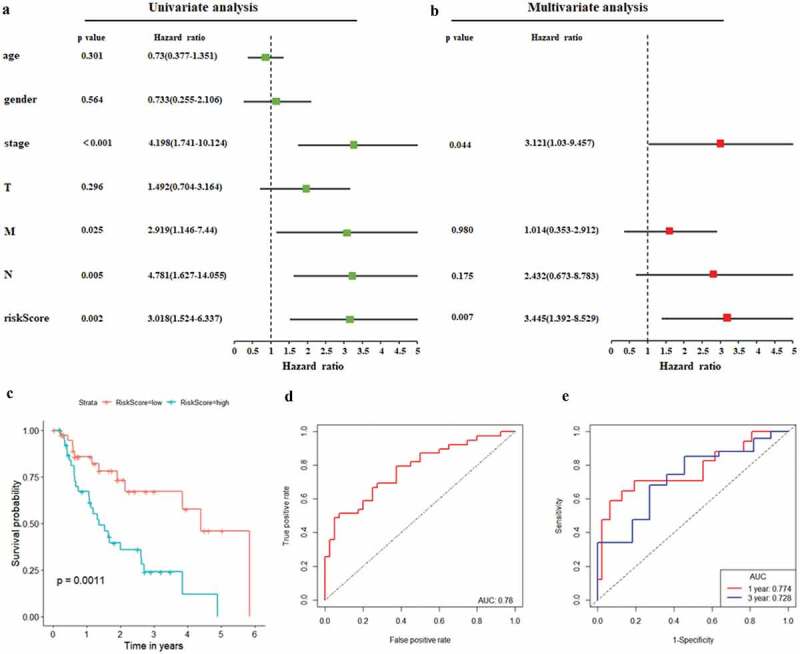
**Autophagy-associated gene signature was significantly associated with EAC survival**. (a) Univariate Cox regression analysis. Forest plot of associations between risk factors and the survival of EAC. (b) multivariate Cox regression analysis. The autophagy-associated gene signature is an independent predictor of TCGA-EAC. (c) Kaplan–Meier analysis of TCGA EAC patients was stratified by median risk. High risk scores are associated with general poor survival of TCGA-EAC. (d) ROC curve of OS-related prognostic model. (e) ROC curves were used to assess the efficiency of the risk signature for predicting 1- and 3-year survival

As illustrated in Figure 5a, patients in the low-risk group had a tendency to express protective genes. In contrast, patients in the high-risk group were more likely to express risk genes (Figure 5a). The distributions of risk score of EAC patients and the relationships between survival time and risk score were visualized in Figure 5b. The prognostic value of risk scores was then assessed. For TCGA-EAC, univariate analysis indicated that risk scores were remarkably associated with OS (HR = 3.018, 95% CI = 1.524–6.337, P = 0.002) (Figure 6a). The risk score and stage were also identified as independent prognostic predictors, as evidenced by multivariate analysis (risk score: HR = 3.445, 95% CI = 1.392–8.529, P = 0.007; stage: HR = 3.121, 95% CI = 1.03–9.457, P = 0.044) (Figure 6b). Kaplan–Meier cumulative curves revealed that patients with low-risk score had longer survival time than those with high-risk score (Figure 6c). The ROC curve of the predictive model was demonstrated in Figure 6d, with AUC of 0.78. In addition, the 1-year and 3-year ROC curves were plotted for TCGA-EAC patients, as shown in Figure 6e.

**Figure 7.**
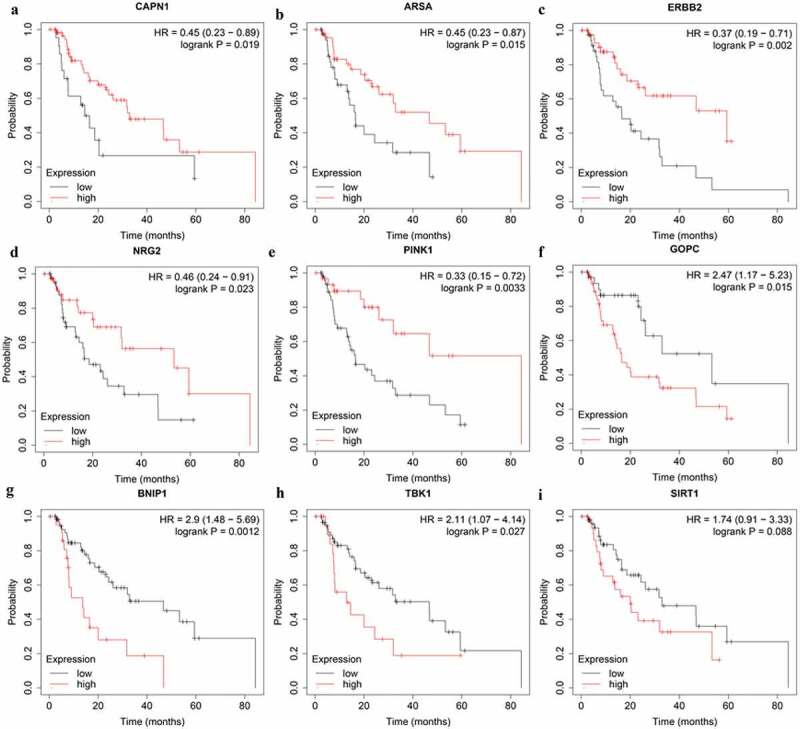
The correlation between ARGs included in OS-related prognostic signature and EAC patients’ survival

Among the nine genes in prognostic prediction model, high expression of CAPN1 (HR = 0.45, 95% CI = 0.23–0.89, P = 0.019), ARSA (HR = 0.45, 95% CI = 0.23–0.87, P = 0.015), ERBB2 (HR = 0.37, 95% CI = 0.19–0.71, P = 0.002), NRG2 (HR = 0.46, 95% CI = 0.24–0.91, P = 0.023), and PINK1 (HR = 0.33, 95%CI = 0.15–0.72, P = 0.0033) genes showed a better OS according to the online Kaplan–Meier Plotter tool (http://kmplot.com/analysis/) (Figure 7a-e). In addition, EAC patients with high expression of GOPC (HR = 2.47, 95% CI = 1.17–5.23, P = 0.015), BNIP1 (HR = 2.9, 95% CI = 1.48–5.69, P = 0.0012), and TBK1 (HR = 2.11, 95% CI = 1.07–4.14, P = 0.027) genes suffered poor prognosis (figure 7f-h). Notably, high SIRT1 expression also were associated with worse prognosis in EAC patients, although not statistically significant (HR = 1.74, 95% CI = 0.91–3.33, P = 0.088) (Figure 7j). Further studies are warranted to investigate the role of SIRT1 in EAC.

### The relationships between clinicopathological parameters and prognosis‑related prediction model

**Figure 8.**
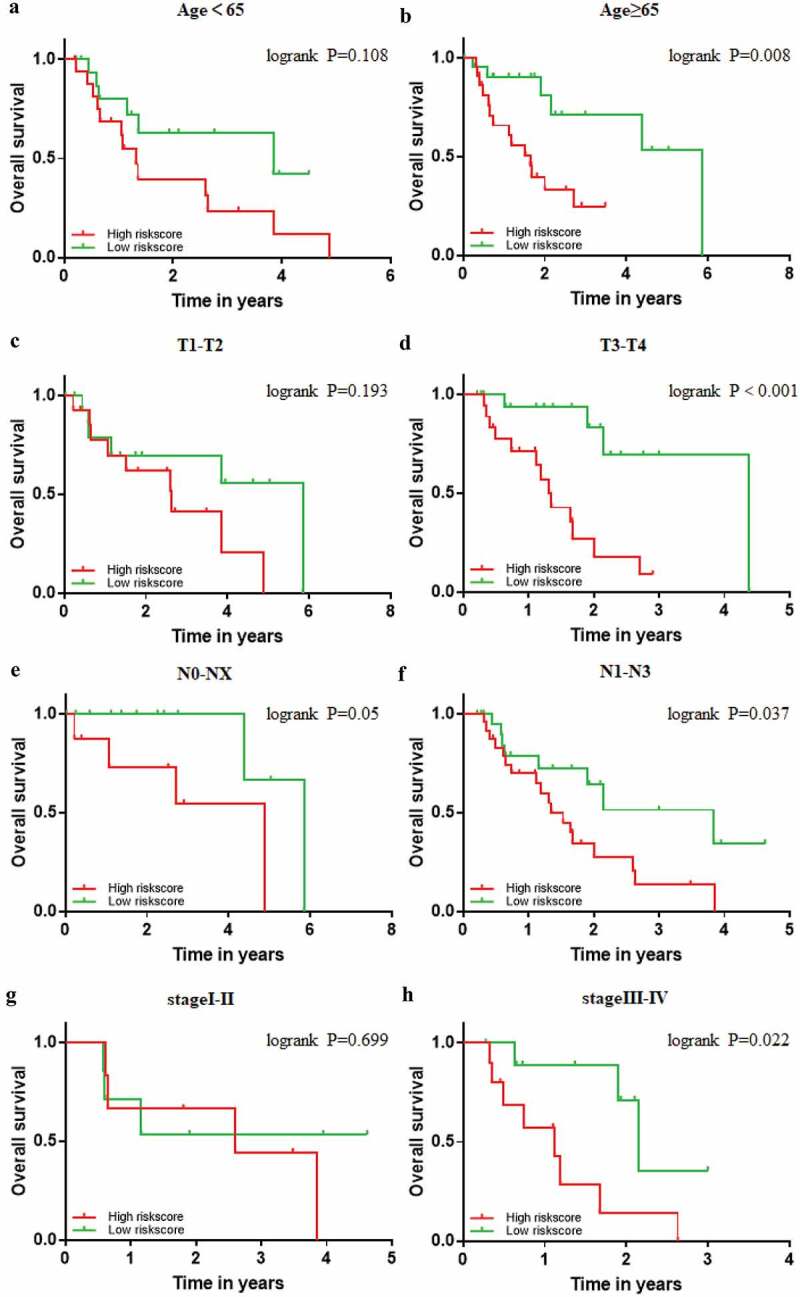
**Confirmation of the signature stratified by different clinical factors in the TCGA EAC cohort**. Kaplan–Meier survival for OS in subgroups stratified by age<65 (a) age≥65 (b) T1,2(c) T3,4(d) N0,X (e) N1,2,3 (f) stage I,II (g) stage III,IV (h) in the TCGA cohort

The prognostic power of the 9-gene signature was further assessed in different subgroups of clinicopathological parameters. Kaplan–Meier cumulative curves showed that EAC patients with high-risk score had worse outcome in different subgroups of clinicopathological parameters, which is consistent with the above result (Figure 8). Despite no statistical difference in groups of T1-T2 and stage I-II, EAC patients with high-risk score also exhibited poor prognosis (Figure 8). On account of most EAC patients were diagnosed in the advanced stages, it still remains to be investigated when the number of EAC patients in T1-T2 or stage I –II, increases to a certain extent.

### Functional enrichment analysis

**Figure 9.**
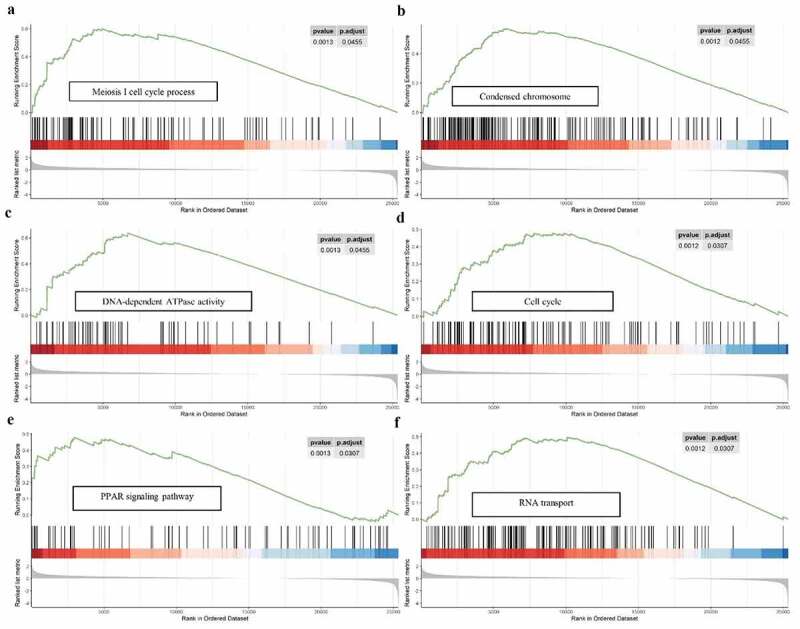
**GSEA analysis of the 9-gene signature between high and low risk groups**. GSEA analysis showed that the high-risk group were significantly associated with meiosis-related processes (a), condensed chromosome (b), DNA-dependent ATPase activity (c), cell cycle (d), PPAR signaling pathway (e), as well as RNA transport (f)

For a more in‐depth understanding the molecular functions and potential mechanisms of 9-autophagy gene signature in EAC, Gene set enrichment analysis (GSEA) was conducted (Figure 9). GSEA analysis revealed that multiple biological processes, especially meiosis-related processes, were enriched in the high-risk group. With respect to cellular components, the high-risk group was dramatically correlated with condensed chromosome. GSEA analysis also demonstrated high-risk group associated with diverse molecular functions, including DNA-dependent ATPase activity, hormone activity, DNA replication origin binding, and so on. In addition, GSEA analysis showed that the high-risk group were dramatically correlated with several common pathways, including cell cycle (NES = 1.72), PPAR signaling pathway (NES = 1.698), as well as RNA transport (NES = 1.841).

### Validation of the prognosis‑related prediction model

**Figure 10.**
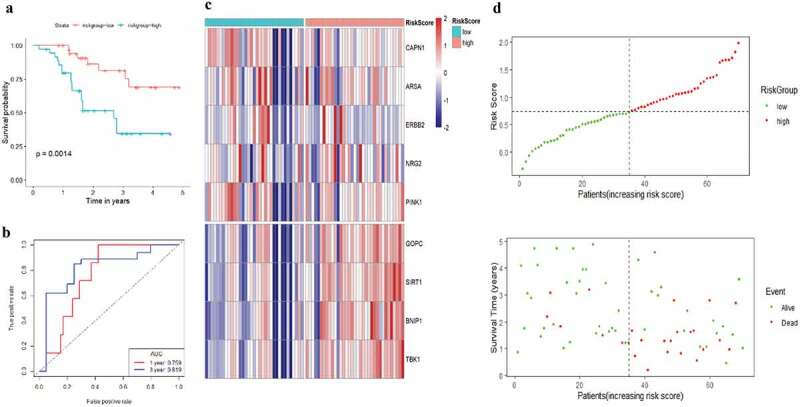
**Risk scores of 9-autophagy gene signature were significantly associated with survival in GSE13898 cohort**. The Kaplan-Meier survival curve (a), ROC curve (b), heatmap (c), and distribution of risk score (d) for the Kim cohort

A total of 70 EAC samples in the Kim data set (GSE13898) in the GEO database were collected and used for validation data set to evaluate the performance of the prognostic gene signature. We separated EAC patients into high- and low-risk groups based on the calculated risk score. The median OS of high-risk patients was 1.62 years, whereas the survival of low-risk patients was 2.18 years. The Kaplan–Meier survival curves indicated that patients in high-risk group had worse prognosis than those in low-risk group (Figure 10a, Kim, P = 0.0014). Following that the ROC curve was plotted to evaluate the accuracy of the model, and the AUCs of ROC curves for predicting 1-, and 3-year survival of EAC in the Kim data set were 0.759 and 0.819, respectively (Figure 10b). The heat map was drawn to exhibit the prognostic gene signature expression profiles in the Kim data set, which was consistent with TCGA-EAC cohort (Figure 10c). Figure 10d showed the risk scores distribution and patients’ survival status in the GEO database. As evidenced by the scatterplot, the mortality rate of EAC patients rose with the increase of risk score (Figure 10d). To sum up, these results confirmed that this 9-autophagy gene signature also performed well for predicting the prognosis of EAC patients in the independent validation EAC cohort.

### Construction and verification of a nomogram for predicting 1- and 3-year survival rate of EAC

**Figure 11.**
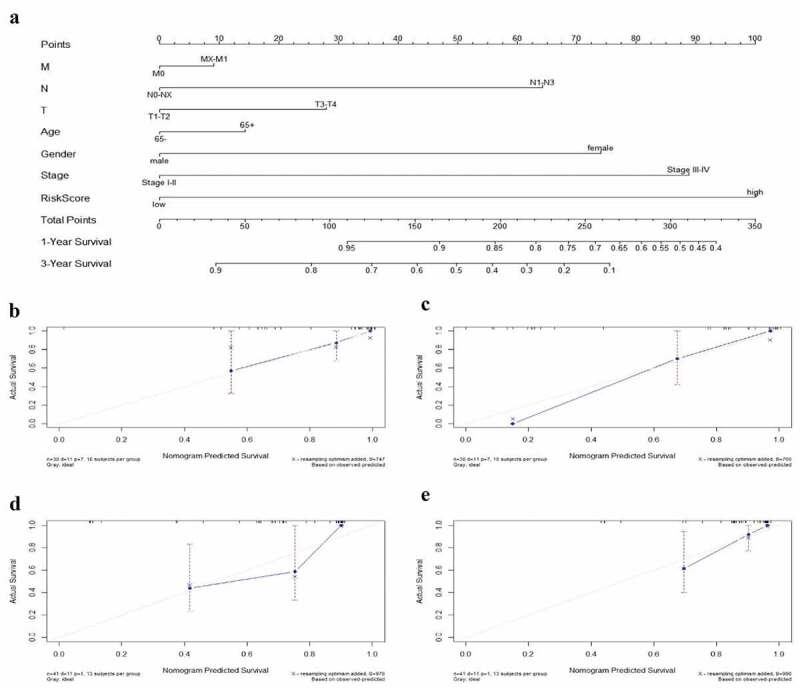
**The nomogram to anticipate prognostic probabilities in TCGA-EAC**. (a) The nomogram for predicting the OS of TCGA-EAC cohort (training set). The above line indicates the risk point for each factor which can be summed up to obtain a overall risk point and the bottom two line indicate the 1-year and 3-year survival possibility. (b–c) The calibration plots for predicting 1-year (b) and 3-year survival (c) in the training set. The calibration plots of 1-year (d) and 3-year survival (e) in the GSE13898 EAC cohort (testing set). The x-axis and y-axis stood for the predicted and actual survival rates of the nomogram, respectively. The solid line indicated the predicted nomogram and the vertical bars represent the 95% confidence interval. The closer the solid line is to the diagonal, the better the prediction

The nomogram has been identified as a robust tool to quantify individuals’ risk by integrating multiple risk factors in the clinical setting [18]. We constructed a nomogram for predicting 1- and 3-year overall survival rate of EAC via incorporating the 9-autophagy gene signature and clinicopathological factors (age, gender, T, N, M, and stage). As shown in Figure 11a, the point was assigned to each risk factor in proportion to its risk contribution to survival, with concordance index (C-index) of 0.74. Moreover, calibration curves showed good concordance between actual and nomogram-predicted survival (Figure 11b and 11c).

The GSE13898 EAC cohort was applied to validate the nomogram, and 1- and 3-year calibration curves were visualized in Figure 11d and Figure 11e, respectively.

### Identification of ERBB2 as a potential therapeutic target in esophageal adenocarcinoma

**Figure 12.**
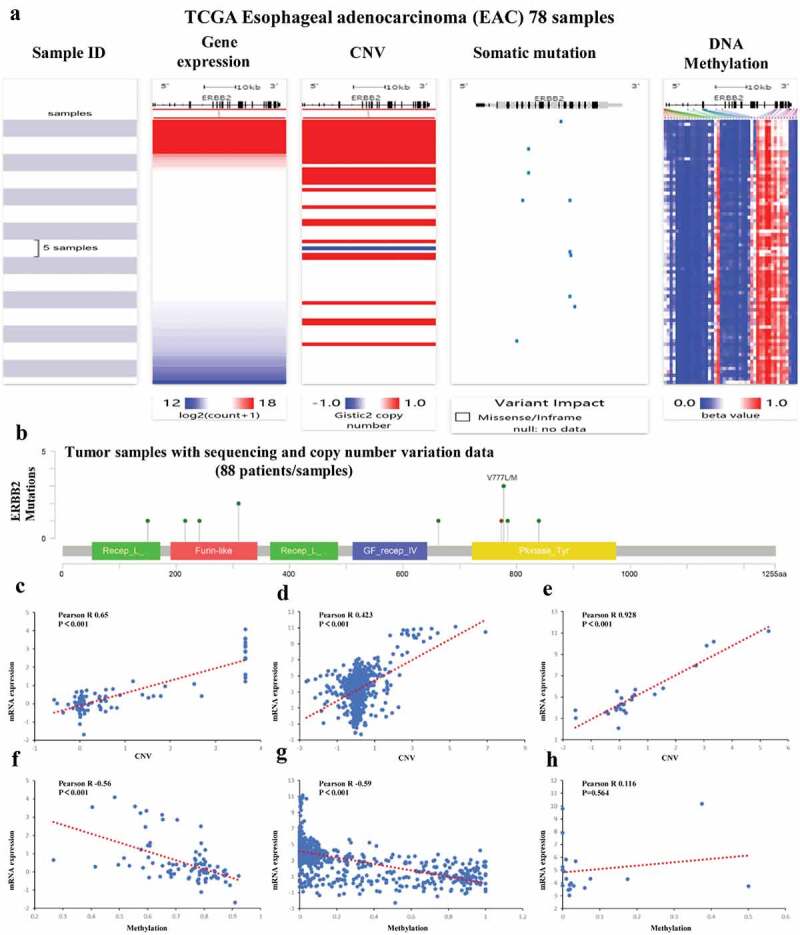
**Mutation, CNV, and methylation analysis of ERBB2 in EAC**. (a) Heatmap showing the correlations between ERBB2 mRNA and CNV, somatic mutations, and methylation in HCC via UCSC Xena. (b) The correlation between ERBB2 mRNA and somatic mutation in EAC. (c) The correlation between ERBB2 mRNA and CNV in EAC. (d) The correlation between ERBB2 mRNA and CNV in 974 cancer cell lines. (e) The correlation between ERBB2 mRNA and CNV in 27 esophageal cancer cell lines. (f) The correlation between ERBB2 mRNA and methylation in EAC. (g) The correlation between ERBB2 mRNA and methylation in 831 cancer cell lines. (h) The correlation between ERBB2 mRNA and methylation in 20 esophageal cancer cell lines. (A) was from UCSC Xena (https://xenabrowser.net/heatmap/), (B), (C), (F) were from the cBioportal for Cancer Genomics (http://www.cbioportal.org/), (D), (E), (G), (H) were from cancer cell line encyclopedia (CCLE) (https://portals.broadinstitute.org/ccle)

Among the nine ARGs in prognostic prediction model, ERBB2 (also known as human epidermal growth factor receptor 2 (Her2)) expression was significantly upregulated in EAC compared with adjacent normal tissues (Figure 3b) and its high expression had a positive correlation with the prognosis of EAC patients (Figure 8). Notably, ERBB2 has a higher mutation rate in EAC than other genes in the prediction model (Figure 4c-d). The potential mechanism of abnormal upregulation of ERBB2 in EAC was further analyzed. As shown in Figure 12a, the UCSC Xena database indicated that the expression of ERBB2 mRNA was associated with CNV and somatic mutations, as well as with several DNA methylation sites. The mutation plot generated by the cBioportal for Cancer Genomics further substantiated the somatic mutations in ERBB2 dysregulation in the 88 included patients/samples (Figure 12b). Moreover, the mRNA expression of ERBB2 exhibited a positive correlation with CNV in 88 TCGA-EAC patients in the cBioportal for Cancer Genomics, as well as in 974 cancer cell lines, and 27 esophageal cancer cell lines in the CCLE database (Figure 12c-e). The mRNA expression of ERBB2 had a significantly negative correlation with DNA methylation in 88 TCGA-EAC patients in cBioportal for Cancer Genomics database and in the whole 831 cancer cell lines but not 20 esophageal cancer cell lines in CCLE database (Figure 12f-h). In conclusion, CNV and DNA methylation might contribute to the dysregulation of ERBB2 in EAC.

**Figure 13.**
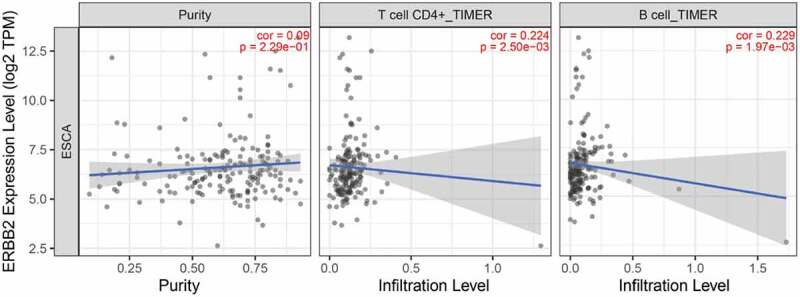
**Correlation of ERBB2 expression with immune infiltration level in the TIMER database**. ERBB2 expression level was positively related to infiltrating levels of CD4 + T cells and B cells

We further analyzed the relationship between ERBB2 expression and tumor infiltrating lymphocytes (TILs) via The Tumor IMmune Estimation Resource (TIMER) algorithm database (https://cistrome.shinyapps.io/timer/) since TILs status played a pivotal role in the progression from esophagitis to Barrett esophagus and EAC [19]. As shown in Figure 13, ERBB2 expression level is positively associated with infiltrating levels of CD4 + T cells (r = 0.224, P = 2.50e-03) and B cells (r = 0.229, P = 1.97e-03). Furthermore, we applied the ENCODE database to predict the TFs that targeted ERBB2 and analyzed the miRNA-ERBB2 interactions using the TarBase and miRTarBase databases (Figure S3A-B). The TF/miRNA-ERBB2 networks may provide new clues for investigation the mechanism of ERBB2 dysregulation in EAC and provide the basis for the development of novel ERBB2-targeted treatments. In addition, the modulation of autophagy genes as potential prognostic biomarkers in EAC should be further researched.

## Discussion

Adenocarcinoma represents the most common histological subtype of EC in many Western countries with incidence rate rising rapidly [4]. Despite diverse genetic drivers and distinct prognostic factors have been broadly explored, EAC patients still suffer from poor survival on account of undetected pathogenesis. Additionally, the present TNM staging system and the existing biomarkers were deemed to insufficient for predicting individual-level prognosis due to lack of sensitivity and specificity [20,21]. It is imperative for clinicians to explore novel methods to predict effectively prognosis and optimize treatment decisions for EAC patients.

Autophagy is a critical catabolic process which functions to maintain cellular homeostasis by degrading dysfunctional cellular macromolecules and organelles in eukaryotic cells [22]. Elevated studies indicated that autophagy was implicated in the esophageal tumorigenesis and a variety of ARGs correlated with the prognosis of EAC patients. The loss of autophagy-related protein Beclin-1 was inversely correlated with histologic grade and tumor stage in EAC patients. Increased Beclin-1 and Beclin-1-phosphorylation expression induced by rapamycin promoted EAC cell survival, suggesting Beclin-1 may serve as a promising marker for EAC survival [23]. Interestingly, selective inhibition of early autophagy induced by siRNA targeted to Beclin 1 significantly enhanced sensitivity of EAC cells to 5-FU, suggesting that specific inhibition of autophagy regulators holds great promises in improving chemotherapeutic regimes [24]. Notably, LC3B globular structures significantly correlate with prognosis of EAC [25]. Moreover, a combination score of nuclear and dot-like/cytoplasmic p62 staining was identified as an independently prognostic parameter for EAC [12]. Given the emerging role of autophagy in EAC, it is promising to speculate that ARGs have great potential in prognostic evaluation. Numerous studies have showed that genomic data, especially multiple-gene signatures, exhibit superior performance in prognostic prediction compared with the current TNM staging system [26,27]. Importantly, prognostic gene signatures based on ARGs have been reported in multiple cancers, such as serous breast cancer, ovarian cancer, colon cancer, and hepatocellular carcinoma [28–31]. For instance, Wang and colleagues recently reported a five-autophagy-related signature (ITGB4, NLRC4, ATG9B, CDKN2A, ERO1A) based on overall survival in patients with lung adenocarcinoma [32].

To our knowledge, the present study is the first to combine the entire set of ARGs with EAC and investigate as well as validate the prognostic value of ARGs in EAC. We determined the mRNA expression of 213 ARGs in the TCGA-EAC dataset. A total of 21 ARGs were identified as differentially expressed genes between 79 EAC and 9 normal tissues. Bioinformatics enrichment analysis demonstrated that 21 differentially expressed ARGs were mainly associated with autophagy, apoptosis, PI3K-AKT signaling pathway, platinum drug resistance, and p53 signaling pathway. Interestingly, Huang et al. research showed that autophagy participated in the regulation of platinum drug resistance of tumor cells [33]. We then performed Cox survival analysis and Lasso regression analysis, constructing a risk model based on nine prognostic ARGs (CAPN1, GOPC, TBK1, SIRT1, ARSA, BNIP1, ERBB2, NRG2, PINK1). The risk scores acquired from the model remarkably stratified patient outcomes in TCGA EAC cohort. The ROC curves and AUCs also suggested that the prognostic prediction model performed well. More importantly, the prognostic performance of the 9-gene signature was validated in an independent GEO EAC dataset (GSE13898). GSEA analysis unveiled that the high-risk group were closely related to cell cycle, PPAR signaling pathway (NES = 1.698), and RNA transport, suggesting that 9-autophagy gene signature may be implicated in the carcinogenesis of EAC by affecting these signaling pathways, thus contributing to a poor prognosis in EAC patients. Notably, accumulating evidence showed that selective autophagy degraded cell cycle proteins, contributing to overcome chemoresistance of tumor cells [34]. Combined targeting of autophagy and cell cycle may serve as a potent anticancer therapeutic strategy for EAC patients. Furthermore, the 9-autophagy gene signature and clinicopathological factors (age, gender, T, N, M, and stage) were integrated to develop a nomogram for predicting 1- and 3-year overall survival rate of EAC. C-index and calibration charts substantiated that the signature could accurately predict the survival of EAC patients. Similar results have been duplicated in an independent GEO EAC cohort (GSE13898).

Interestingly, previous studies have reported that the genes contained in the prognostic signature are all correlated with cancer [35–37]. Of them, TBK1 had attracted our great interests. Not only our study, but Chen et al. and Du et al. also incorporated this gene into their prognosis prediction models based on ARGs in esophageal cancer. Previous study demonstrated that TBK1 exerted a pivotal role in enhancing oncogenic phenotypes and regulating autophagy and mitophagy [38–40]. In addition, TBK1 also takes part in modulating the immune response [41]. Further studies are warranted to investigate the role of TBK1 in EAC. Among the 9-gene signature, SIRT1, PINK1, and ERBB2 have been involved in esophageal carcinogenesis. SIRT1, a NAD+-dependent deacetylase involved in the regulation of DNA repair and metabolism, was considered as an independent survival risk factor in esophageal cancer and its overexpression was associated with worse OS (HR = 1.776, P = 0.009) and disease-free survival (DFS) (HR = 1.642, P = 0.017) [42]. In addition, SIRT1 was related to malignant transformation in drug-resistant esophageal cancer cells [43]. These findings are consistent with our results that SIRT1 was a high-risk gene for EAC. PINK1 expression was upregulated in ESCC patients who underwent chemotherapy and associated with drug resistance as well as poor prognosis for ESCC patients treated with neoadjuvant chemotherapy [44], whereas our results showed that PINK1 was identified as a protective gene and its high expression was associated with favorable prognosis in EAC. Further studies are needed to investigate the role of PINK1 in EAC. As for ERBB2, the present study demonstrates that the dysregulation of ERBB2 in EAC is related to CNV and DNA methylation. And its expression is positively associated with the prognosis of EAC patients and infiltrating levels of CD4 + T cells and B cells in EAC. To our knowledge, TILs were associated with favorable prognosis in patients with EAC [45]. Previous studies also reported that Her2-positive EAC patients may benefit from administration of Her2-targeting monoclonal antibody trastuzumab/Herceptin [3]. Of note, it was reported that autophagy may correlate with acquired resistance to Lapatinib in EAC [46], while ERBB2 was involved in autophagy regulation [47]. Recent study revealed that ERBB2 promoted autophagy via upregulating the autophagy-related 12 (ATG12), which further induced resistance to ERBB2-targeted antibody lapatinib in breast cancer cells [21]. Therefore, we propose that combining Her2-targeted therapy and autophagy inhibition might be beneficial for EAC patients. However, tumor heterogeneity posed a challenge to HER2-targeted therapy in gastroesophageal cancer. Additional endoscopy and biopsy sampling as well as targeted next-generation sequencing (NGS) may be feasible to guide the therapy’s design and could enhance the therapeutic impact of the HER2-targeted treatment in EAC patients [48,49]. Taken together, the combination of ERBB2-based treatment and immunotherapy as well as targeting autophagy exhibits great potential in the management of EAC patients. Further prospective studies are warranted to explore this field.

In summary, our current study profiled the mRNA expression of 213 autophagy-associated genes in the TCGA EAC cohort. We proposed an OS-related prediction model based on 9-autophagy gene signature (CAPN1, GOPC, TBK1, SIRT1, ARSA, BNIP1, ERBB2, NRG2, PINK1), which was an independent prognostic factor for EAC patients. We also constructed a nomogram by incorporating the prognostic gene signature and clinicopathological factors, which had good performance in predicting the OS of EAC patients. In-depth studies of these hub genes may contribute to personalized therapy of esophageal adenocarcinoma in the clinical setting.

## Data Availability

All data generated or analyzed during this study are included in this article. The TCGA data portal (https://portal.gdc.cancer.gov/); the GSE13898 dataset (https://www.ncbi.nlm.nih.gov/geo/query/acc.cgi?acc=GSE13898).
